# Overlooked Population: Physical Health Care for Young, Non-Binary, and Rural Patients With Severe Mental Illness

**DOI:** 10.1192/bjo.2025.10095

**Published:** 2025-06-20

**Authors:** Julia Kim, Russell Roberts, Sharon Lawn

**Affiliations:** ^1^College of Medicine and Public Health, Flinders University, Adelaide, Australia; ^2^School of Business, Charles Sturt University, Barthurst, Australia; ^3^Equally Well, Melbourne, Australia; ^4^College of Medicine and Public Health, Flinders University, Adelaide, Australia; ^5^Lived Experience Australia, Melbourne, Australia

## Abstract

**Aims:** Individuals with severe mental illness (SMI) experience significant physical comorbidities, contributing to a 20-year gap in life expectancy compared with the general Australian population. However, disparities persist in how their physical healthcare needs are addressed in healthcare settings. Recent full report from Unequally Unwell (2024) identifies age, gender and rurality as contributing factors to mortality in SMI patients, yet little research has examined how these factors influence access to physical healthcare. This study investigates whether age, gender and rurality predict healthcare engagement, specifically whether patients access their care and whether their physical health is addressed in general practitioner (GP) and psychiatric consultations.

**Methods:** This study analysed a de-identified dataset from 235 mental health patients and 96 carers who participated in a survey conducted by Lived Experience Australia. Participants provided demographic information and reported on their healthcare experiences, including whether they had visited a GP or a psychiatrist in the past 12 months, and whether their physical health was discussed. Chi-square tests and ordinal logistic regression were used to assess relationships between these variables.

**Results:** Results showed that age significantly influenced whether a psychiatrist inquired about physical health, with older individuals being more likely to be asked (B=0.879, SE=0.431, z=2.039, OR=2.41, p=0.041). No significant associations were found between rurality and healthcare engagement, though a non-significant trend suggested potential disparities. Similarly, gender identity did not significantly predict physical health discussions, though a weak-to-moderate association was observed for psychiatrist visits (χ²(2)=8.03, p=0.018); an effect which disappeared when non-binary individuals with SMI were removed from the analysis. Notably, engagement with GPs showed no significant differences across demographic groups.

**Conclusion:** These findings suggest that younger individuals with SMI may be at a greater risk of diagnostic overshadowing for psychiatric care, where their physical health concerns are overlooked. Additionally, non-binary individuals with SMI appear to have lower rates of psychiatric utilisation within 12 months. This disparity may be influenced by systemic barriers such as stigma and discrimination in healthcare settings, which can lead to decreased help-seeking behaviours or suboptimal care experiences for non-binary individuals. This highlights the need for targeted interventions to ensure routine physical health discussion/screening within mental health services, which could facilitate early detection of physical health issues, and help mitigate the mortality gap in this vulnerable population.

